# The impacts of stress and loneliness on gambling and gaming problems: A nationwide longitudinal study

**DOI:** 10.1177/00207640241264661

**Published:** 2024-07-25

**Authors:** Ilkka Vuorinen, Iina Savolainen, Anu Sirola, Atte Oksanen

**Affiliations:** 1 Tampere University, Finland; 2 University of Jyväskylä, Keski-Suomi, Finland

**Keywords:** Gambling problems, gaming problems, loneliness, stress, addiction

## Abstract

**Background:**

Problems related to gambling and digital gaming have been a topic of concern for years. Less attention has been paid to the probable psychosocial factors behind these problems. While previous studies have established links between stress, loneliness, and addiction, there is a lack of longitudinal research investigating how stress and loneliness affect addictive behaviors, including problem gambling and gaming.

**Aims:**

This study uses multilevel mixed-effects generalized linear models to analyze the between- and within-person effects of stress and loneliness on gambling and gaming problems. The interaction between stress and loneliness was also investigated.

**Methods:**

A representative sample of Finns (*N* = 1,530) answered a survey in 6-month intervals between spring 2021 and autumn 2023; 49.22% of the sample took part at all six time points. The Problem Gambling Severity Index and the Internet Gaming Disorder Test were used to measure gambling and gaming problems. The three-item UCLA Loneliness Scale was used to assess loneliness, and the Perceived Stress Scale was used to evaluate stress.

**Results:**

After controlling for gender and age, loneliness was found to increase only gaming problems at both the between- and within-person levels, but not gambling. In contrast, stress enhanced both gambling and gaming problems at the between- and within-person levels. Additionally, loneliness and stress were found to have negative interaction suggesting that their combined effect was lower than their separate effects.

**Conclusion:**

The findings provide longitudinal insight into the psychosocial vulnerabilities behind problem gambling and gaming, which can be helpful in designing targeted interventions.

## Introduction

People play digital games and gamble sometimes to the extent that problems emerge. This has raised concerns in Finland, where different forms of gambling are widely available. In the country, 78.4% of those aged 15 to 74 years gamble at least once a year, and 29% of the same population do so at least once a week (Salonen et al., 2020). In comparison, digital games are played at least sometimes by 80.3% of those aged 10 to 75 years, and entertainment games are played at least once a week by 41.1% of this population (Kinnunen et al., 2022). While gambling is typically something that revolves around money or other stakes, digital games are usually story- or challenge-driven pastimes. However, technological advancements have blurred the distinction between these two activities, and monetary and gambling-like features are now common revenue models in digital games; also, gambling games increasingly utilize narrative elements adapted from video games (Gainsbury et al., 2014; Macey & Hamari, 2022).

As pastimes, gambling and playing digital games do not necessarily cause problems. However, spending excessive amounts of time and money on these games can. This has been recognized even in the DSM-V as a conditions that can lead to many clinically significant issues relating to mental health, preoccupation, and jeopardized social obligations (American Psychiatric Association, 2013). As with all potentially addictive behaviors, gambling and gaming problems are more likely to accumulate among individuals who are somehow more vulnerable, such as those who have lower socioeconomic status (Kochuchakkalackal et al., 2020; Sharman et al., 2019). In addition to other mental health conditions associated with problem gambling and gaming (e.g. emotion regulation, anxiety, and mental distress; Marchica et al., 2019; Savolainen et al., 2022), it is important to investigate how stress and loneliness impact the vulnerability to these problems, especially as loneliness rates are increasing and people encounter a multitude of stressors in their lives.

Stress is a physical response that is triggered when the body’s homeostasis is threatened by either an actual or a perceived threat (Chrousos, 2009). Stress can be acute or chronic. Acute stress involves a response to a specific stimulus or situation. Once the stressor is removed, the body returns to its normal state. Chronic stress persists over a longer period, stemming, for example, from difficult life situations or enduring hardships. Elevated levels of stress hormones can result in a wide array of negative symptoms and health issues (Russell & Lightman, 2019). Stress is known to accompany addictive behaviors. For instance, it can contribute to and worsen as a result of alcohol or drug addiction (Ruisoto & Contador, 2019), and it can increase the risk of relapse (Roche et al., 2017). Stress has also been found to contribute to problem gambling and gaming through coping motives (Maroney et al., 2019; Thomas et al., 2011), which suggests that these behaviors are sometimes used as ways to cope with life stressors. Perceived stress has also been shown to influence consumer behavior. According to a study of gambling and gaming during the COVID-19 pandemic, perceived stress intensified the association between spending money on gambling or within digital games and gambling or gaming problems (Savolainen et al., 2023).

Loneliness is a painful experience of perceived discrepancy between one’s desired and actual levels of social connection (Perlman & Peplau, 1981). It is often divided into social and emotional loneliness; the former refers to a lack of social connections in general, while the latter pertains to a lack of meaningful and close relationships (Weiss, 1973). Loneliness has crucial clinical significance due to its association with many well-being deficits and even premature death (Park et al., 2020). As social animals, humans have a psychological need to belong (Baumeister & Leary, 1995; Ryan & Deci, 2017), and addiction is often associated with challenges in normal social interactions (Alexander, 2008; Heilig et al., 2016; Rachlin, 2000).

Several studies have investigated how loneliness is related to gambling and gaming. While the condition is a risk factor for gaming problems (Kochuchakkalackal et al., 2020), the available cross-sectional evidence provides mixed results on this relationship (Nordmyr & Forsman, 2020), suggesting that its causal associations are complex. The literature has shown that anxiety and loneliness are associated with problem-gambling behavior, particularly during youth (Savolainen et al., 2020) and at an older age (Parke et al., 2018). Loneliness can also lead to mental distress and thus more intense gambling problems (Vuorinen et al., 2021). One experimental study found that lonely individuals were more likely to show interest in gambling content in online settings (Sirola et al., 2019). Qualitative research also indicates that loneliness is a reason for gambling (Nordmyr & Forsman, 2020). However, there is a need for longitudinal evidence in this area.

Given the complexities among psychosocial variables, perceived stress and loneliness are likely to coexist. Chronic loneliness can increase stress responses (Cacioppo et al., 2015), and studies have shown that higher loneliness predicts exaggerated stress responses when dealing with acute stressors (Brown et al., 2018). Lonely people can also perceive social situations as stressful and threatening (Nowland et al., 2018), which may be due to underlying deficits in social skills (Segrin, 2019). Indeed, it has been hypothesized that stress tendency can be one underlying causal factor for feelings of loneliness (Campagne, 2019). Thus, stress and loneliness are likely to have a close reciprocal link.

This study investigated the impacts of stress and loneliness on problems associated with gambling and digital gaming. Its main hypotheses were the following: (1) Perceived loneliness increases gambling and gaming problems; (2) perceived stress increases gambling and gaming problems; and (3) gambling and gaming problems are further increased by the combination of stress and loneliness. Based on the available literature, it is to be expected that stress and loneliness intensify gambling and gaming problems, but the dynamic relationships in question have not been previously explored in longitudinal population-wide settings.

## Methods

### Participants

This study used data from the longitudinal six-wave *Gambling in the Digital Age* survey, which was collected in 6-month intervals between spring 2021 and autumn 2023. The initial data were collected in April 2021 from a panel of Finnish volunteers aged 18 to 75 years by Norstat Finland, a data-provider company, upon request by the research group. The response rate at the first collection point was 34.60% (*N* = 1,530; *M*_age_ = 46.7 years; 50.33% men). Each subsequent data collection point had some loss of participants (*N*_T2_ = 1,198; *N*_T3_ = 1,095; *N*_T4_ = 1,004; *N*_T5_ = 934; *N*_T6_ = 889; respectively); despite this, the overall data remained robust in its demographic representativeness, both internally and compared to the Finnish adult population. In the end, 753 participants (49.22%) took part in all the survey waves. Each survey took approximately 15 minutes to complete.

Several important steps were made to ensure the ethical quality of the study based on the Declaration of Helsinki. First, the Academic Ethics Committee of the Tampere region approved the study before the start of data collection. Second, the participants were informed of the purpose of the research at the beginning of the survey and could withdraw from the study at any time without consequences. The completion of the full survey was taken as a consent for participation. Third, Norstat Finland provided only anonymized data to the research group. Fourth, the researchers conducted quality checks following a preestablished protocol to detect and remove participants with consistently or logically biased response patterns.

### Measures

This study had two outcome variables. Gambling problems were measured with the Problem Gambling Severity Index (PGSI; Ferris & Wynne, 2001). The PGSI consists of nine items, which measure different kinds of gambling harms, as indicated in the DSM-V (American Psychiatric Association, 2013), using a 4-point scale from 0 (never) to 3 (almost always). All the items of the PGSI were combined to form a scale from 0 to 27. As most of the participants had no gambling problems, the resulting scale had a very low mean and was highly skewed to the right. The omega (ω) coefficients ranged from .94 to .95 at all time points, which indicates excellent internal reliability.

Gaming problems were measured with the Ten-Item Internet Gaming Disorder Test (IGDT-10; Király et al., 2017). Its 10 items measure various gaming harms from preoccupation to playing despite negative consequences, and they use a 3-point scale ranging from 0 (never) to 2 (often). Following the scoring guide developed by Király et al. (2017), all the items were coded in binary form, with only the answer ‘often’ giving 1 point and items 9 and 10 giving a maximum of 1 point. Thus, the total score ranged from 0 to 9. As was the case for the PGSI, most of the participants had no gaming problems, which resulted in a low mean and high right skewness. The ω coefficients ranged from .87 to .88, indicating high internal reliability.

Stress was measured with the Perceived Stress Scale (PSS; Cohen et al., 1983; Cohen & Williamson, 1988). The PSS is a 10-item scale that measures the experience of stress in the past month via questions such as ‘How often have you felt that difficulties were piling up so high that you could not overcome them?’ The answers were provided on a 5-point scale ranging from 0 (never) to 4 (very often). When the items were combined, the total score ranged from 0 to 40. The ω coefficients ranged from .88 to .89, which indicates good internal consistency across the time points.

Loneliness was measured with the 3-item UCLA Loneliness Scale (Hughes et al., 2004). This is a short version of a much wider loneliness questionnaire, and it uses a 3-point scale from 0 (hardly ever) to 2 (often). This short measure has been shown to efficiently capture different aspects of perceived loneliness in survey studies. The total scores ranged from 0 to 6. The ω coefficients ranged from .84 to .86.

In addition to the measures above, age and gender were included as background variables. Gender was transformed into a binary variable to compare men (1) to women and other genders (0). The descriptive statistics can be found in Table 1.

**Table 1.** Descriptive statistics.

Variables	Range	*M* (SD), T1	*M* (SD), T2	*M* (SD), T3	*M* (SD), T4	*M* (SD), T5	*M* (SD), T6
PGSI	0–25	1.31 (3.33)	1.18 (3.15)	1.18 (3.18)	1.06 (2.93)	1.00 (2.80)	.89 (2.63)
IGDT	0–9	.15 (.69)	.12 (.61)	.08 (.49)	.09 (.59)	.08 (.45)	.10 (.58)
PSS	0–40	13.61 (7.04)	13.43 (6.95)	13.68 (6.87)	13.25 (6.81)	12.88 (6.96)	12.72 (6.72)
UCLA-LS	0–6	1.76 (1.77)	1.74 (1.70)	1.77 (1.71)	1.71 (1.71)	1.60 (1.67)	1.53 (1.67)
Age	18–75	46.67 (16.42)	48.87 (16.11)	49.72 (16.16)	50.73 (15.90)	51.91 (15.42)	53 (15.27)
		*N*_T1_ (%)	*N*_T2_ (%)	*N*_T3_ (%)	*N*_T4_ (%)	*N*_T5_ (%)	*N*_T6_ (%)
Male	0/1	770 (50.33)	608 (50.75)	548 (50.05)	506 (50.40)	472 (50.54)	447 (50.28)

### Statistical analyses

The analyses were conducted with the software Stata 18 (StataCorp). Instead of Cronbach’s alpha, the more refined McDonald’s ω (*omegacoef* command) was employed to measure the reliability of the scales (Hayes & Coutts, 2020).

For the main analyses, hybrid multilevel-regression models were run with the *xthybrid* command, which is based on generalized linear mixed modeling (Schunck & Perales, 2017). These kinds of hybrid models are helpful in analyzing both the within-person (changes within an individual) and between-person (average differences between individuals) effects of time-varying independent variables on time-varying dependent variables as they combine the advantages of both fixed- and random-effects models with more flexible estimation (Schunck, 2013; Schunck & Perales, 2017). The skewed distributions of the dependent variables were taken into account by choosing a negative binomial family with a log link and robust standard errors, which are commonly used in similar circumstances (Baggio et al., 2018). Only those participants (*n* = 753) who answered at all time points were included in the analyses. The model assumptions were checked by obtaining the VIF scores from the linear-regression models at different time points.

The *xtnbreg* command was used to create random-effects overdispersion models of the interaction between stress and loneliness. By doing so, the models could still consider skewed dependent-variable distributions while allowing the use of interaction terms, unlike hybrid models. To avoid potential issues, the loneliness and stress variables were standardized.

## Results

The correlation matrix (Table 2) shows that almost all the variables were significantly correlated. Gambling and gaming problems had low-to-moderate correlations with the independent variables. The highest correlation was between stress and loneliness (*r* = .62), but age had also relatively high correlations with stress (*r* = −.31) and loneliness (*r* = −.22). Stress was more strongly correlated with gambling problems (*r* = .23) than gaming problems (*r* = .19), but loneliness had the same correlation with both (*r* = 18).

**Table 2.** Correlation matrix (T1).

Variables	1.	2.	3.	4.	5.	6.
1. PGSI	1					
2. IGDT	.47***	1				
3. PSS	.23***	.19***	1			
4. UCLA-LS	.18***	.18***	.62***	1		
5. Age	−.16***	−.15***	−.31***	−.22***	1	
6. Male	.07***	.05*	−.11***	−.08**	−.03	1

* *p* < .05. **p < .01. ***p < .001.

Table 3 shows the effects of loneliness and stress on gambling and gaming problems. The incidence-rate ratios (IRRs) indicate that every unit increase in perceived loneliness multiplied gaming problems by 1.10 at the within-person level and by 1.16 at the between-person level. In contrast, loneliness had no significant effect on gambling problems. Stress multiplied both gambling and gaming problems by 1.02 at the within-person level, but at the between-person level, it multiplied gambling problems by 1.18 and gaming problems by 1.10 per every unit increase.

**Table 3.** Hybrid models of the effects of loneliness and stress on gambling problems and gaming problems. Only those who participated in all survey waves are included.

Variables	Gambling problems			Gaming problems		
	*B*	Robust *SE*	IRR	*B*	Robust *SE*	IRR
Within-person variables						
Loneliness	0.05	0.03	1.05	0.10***	0.03	1.10
Stress	0.02*	0.01	1.02	0.02**	0.01	1.02
Between-person variables						
Loneliness	0.04	0.08	1.04	0.15*	0.07	1.16
Stress	0.17***	0.02	1.18	0.09***	0.02	1.10
Age	−0.03***	0.01	0.97	−0.06***	0.01	0.95
Gender (M:1)	0.714***	0.17	2.04	0.44**	0.14	1.55
/lnalpha	−1.49	.29	.23	−1.35	.23	.26
Random part						
Variance (constant)	7.19	0.63	1322.71	3.50	0.28	33.19

* *p* < .05. **p < .01. ***p < .001.

Age and gender were included as background variables to control for their impacts. Both had statistically significant effects on the outcome variables. While every increase in age lowered gambling problems by a multiplier of .97 and gaming problems by a multiplier of .95, male gender increased the former by 2.04 and the latter by 1.55. Thus, male gender had the highest effect on both outcome variables, even though, as a binary variable, the effect was limited.

Interaction models were run to verify the presence of interaction between the random effects of loneliness and stress. These models (Table 4) revealed significant interaction between the two variables regarding both gambling and gaming problems. For both models, the interaction term was negative, with a multiplier of .94. This means that the combined effect of loneliness and stress can be expected to be slightly lower than when the effects are analyzed separately. The standardized IRR multipliers for loneliness were 1.14 when gambling problems were the outcome variable and 1.27 when gaming problems were the outcome variable. Similarly, the standardized IRRs for stress were 1.26 concerning gambling problems and 1.24 concerning gaming problems. The background variables had fairly similar effects in these models as in the hybrid ones, with the exception of age, which lost its significance as a predictor of gambling problems.

**Table 4.** Interactions between loneliness and stress, random effects.

Variables	Gambling problems			Gaming problems		
	*B*	Robust *SE*	IRR	*B*	Robust *SE*	IRR
Loneliness (standardized)	0.13**	0.05	1.14	0.24***	0.04	1.27
Stress (standardized)	0.23***	0.05	1.26	0.21***	0.04	1.24
Loneliness × stress	−0.06*	0.03	0.94	−0.06**	0.02	.94
Age	0.00	0.01	1.00	−0.04***	0.00	0.96
Gender (M:1)	0.77***	0.36	2.16	0.34**	0.12	1.40
Random part						
/ln_*r*	.53	0.12		0.96	0.11	
/ln_*s*	−1.72	0.08		−0.92	0.08	
*r*	1.70	.20		2.62	.29	
*s*	0.18	0.01		0.40	0.03	

* *p* < .05. **p < .01. ***p < .001.

## Discussion

This study investigated how stress and loneliness impact gambling and gaming problems. The hypotheses were based on the assumption that stress and loneliness increase these problems separately and cumulatively. Hybrid multilevel-regression models were created to evaluate separate effects, while random-effects overdispersion models were run to check for the interaction between loneliness and stress. Based on the results, the first hypothesis was only partially supported, since perceived loneliness increased only gaming problems, significantly. Regarding the second hypothesis, perceived stress enhanced both gambling and gaming problems. Finally, contrary to the third hypothesis, the interaction models revealed that the combined effect of stress and loneliness was in fact lower than their separate effects.

Psychological suffering is at the core of addictions, and the associations between these two phenomena are usually quite complex and multifaceted. On its own, stress increased both gambling and gaming problems. The same was true of loneliness, but only as a predictor of gaming problems. These findings are in line with the literature, where stress has been associated with gambling and gaming problems (Maroney et al., 2019; Thomas et al., 2011). Loneliness has also been recognized as a risk factor for these problems, although with a more complicated causal relationship (Kochuchakkalackal et al., 2020; Nordmyr & Forsman, 2020; Vuorinen et al., 2021). Both of these factors are tied to individual life circumstances and might thus change when people’s lives change. For instance, loneliness and problem gambling are particularly associated among the elderly (Parke et al., 2018). Older people playing slot machines is a common sight in Finland, and since the results of this study indicate that aging reduces problem gambling, focusing on their psychological and social well-being could help to reduce this behavior among those who appear to play excessively.

The negative interaction presented in this study can have multiple explanations. A practical one could be that people who are both stressed and lonely enough may simply not have the motivation to play. Based on the high correlation between stress and loneliness, another explanation could be that these two conditions coexist to the extent that they are partly the same phenomenon. This would also be in line with the literature that links stress with loneliness (Cacioppo et al., 2015; Campagne, 2019; Nowland et al., 2018). It is also possible that there is a confounding factor that was not included in the present study. For example, stress has been found to be indirectly associated with gambling and gaming problems and loneliness with gaming problems through different coping mechanisms (Maroney et al., 2019; Melodia et al., 2022; Thomas et al., 2011).

### Limitations

Some potential limitations of our study should be considered. Although the survey data matched the population demographics well, the response rate for the first survey was only 34.6%, which means that the study could have attracted people who were interested in gambling as a topic. Despite this, the number of those who reported having experienced gambling or gaming problems was only slightly higher than the official national estimates found in Salonen et al. (2020). Furthermore, as the survey was based on self-reports, the answers might have varied depending on people’s interpretations, the underreporting of socially undesirable behaviors (e.g. gambling), and the reliance on memory when answering.

### Implications and future research

People have many reasons to play games or gamble, and problems related to these activities are tied to a complex array of factors. This longitudinal study approached gambling and gaming addictions based on the assumption that the wider psychosocial issues of perceived stress and loneliness could increase such addictions. The results suggest that these issues contribute to increasing gambling and gaming problems over time, although some differences exist in their impacts and people who experience both loneliness and stress might not have the energy to play or gamble at the population level. Clinical samples could be utilized in future studies to investigate the interaction of these factors among people who have experienced gambling or gaming addiction. Nevertheless, it would be beneficial for society to target the factors that cause stress and loneliness as a preventive measure to reduce these forms of addiction.

## Conclusions

In this study, stress and loneliness were hypothesized to increase gambling and gaming problems both separately and in conjunction. While they did mostly increase these problems, loneliness enhanced only gaming addiction, and the interaction effect of stress and loneliness was lower than their separate effects. This study contributes to our understanding of how these factors influence problem gambling and gaming by investigating their interaction at the population level and revealing slight differences in how they affect gambling and gaming addictions. Although gambling and digital-gaming behaviors are considered problematic when performed in excess, it is necessary to look for wider psychosocial factors that might aggravate this situation and ensure that people feel well in their everyday lives.
